# Determinants of Host Range Specificity in Legume-Rhizobia Symbiosis

**DOI:** 10.3389/fmicb.2020.585749

**Published:** 2020-11-27

**Authors:** Liam Walker, Beatriz Lagunas, Miriam L. Gifford

**Affiliations:** ^1^School of Life Sciences, University of Warwick, Coventry, United Kingdom; ^2^Warwick Integrative Synthetic Biology Centre, University of Warwick, Coventry, United Kingdom

**Keywords:** specificity, rhizobia, legume, host range, symbiosis, nodulation

## Abstract

Leguminous plants possess the almost unique ability to enter symbiosis with soil-resident, nitrogen fixing bacteria called rhizobia. During this symbiosis, the bacteria physically colonize specialized organs on the roots of the host plant called nodules, where they reduce atmospheric nitrogen into forms that can be assimilated by the host plant and receive photosynthates in return. In order for nodule development to occur, there is extensive chemical cross-talk between both parties during the formative stages of the symbiosis. The vast majority of the legume family are capable of forming root nodules and typically rhizobia are only able to fix nitrogen within the context of this symbiotic association. However, many legume species only enter productive symbiosis with a few, or even single rhizobial species or strains, and vice-versa. Permitting symbiosis with only rhizobial strains that will be able to fix nitrogen with high efficiency is a crucial strategy for the host plant to prevent cheating by rhizobia. This selectivity is enforced at all stages of the symbiosis, with partner choice beginning during the initial communication between the plant and rhizobia. However, it can also be influenced even once nitrogen-fixing nodules have developed on the root. This review sets out current knowledge about the molecular mechanisms employed by both parties to influence host range during legume-rhizobia symbiosis.

## Introduction

The legume family is almost unique amongst plants in that its members are able to interact with soil dwelling bacteria called rhizobia. This leads to nitrogen-fixing symbiosis, during which specialized structures called root nodules form on the plant root. These nodules are colonized by the rhizobia, then reduce atmospheric nitrogen to ammonia, in a process known as biological nitrogen fixation (BNF). This fixed nitrogen is utilized by the host plant for growth whilst photosynthates, in the form of dicarboxylic acids, are provided to the rhizobia as a carbon source in exchange (White et al., 2007).

Nitrogen (N)-fixing symbiosis begins with molecular cross-talk between the plant root and rhizobia. During times of nitrogen paucity, polyphenolic compounds called flavonoids are exuded by the root into the rhizosphere. These compounds are able to diffuse across the membranes of rhizobia in their vicinity (Fisher and Long, 1992). Upon flavonoid perception, rhizobia respond by activating transcription of symbiosis-related (Nod) genes and are chemoattracted into closer proximity to the plant root. Nod gene expression is orchestrated by the activity of nodulation protein D (NodD) binding to the *nod* box found upstream of these genes. The key effect of expression of Nod genes is the production and secretion of lipochitooligosaccharide (LCO) compounds called Nod factors (Schlaman et al., 1992). Some rhizobia possess multiple copies of *nodD* (Perret et al., 2000) and/or a copy of a repressor of *nod* gene expression called *nolR* (Kiss et al., 1998).

Nod factors are then responsible for driving the host plant toward symbiosis. They are recognized by membrane-localized proteins called Nod factor receptors, leading to root hair deformation and activation of nodulation-related genes. Nod factor perception is mediated by co-receptors NFR1/NFR5 in *Lotus japonicus* (Radutoiu et al., 2003) and LYK3/NFP in *Medicago truncatula* (Amor et al., 2003; Smit et al., 2007). The exact processes that occur as a consequence of this can differ substantially between different legume-rhizobia partnerships; for a detailed overview of this process, see (Gage, 2004; Sprent, 2009). Most commonly, localized inhibition of growth at the tips of root hair cells induces curling of root hairs, creating a pocket in which rhizobia may become trapped (Esseling et al., 2003). Localized cell wall degradation (Xie et al., 2012), cytoskeletal re-arrangement and vesicle trafficking give rise to a tubular cell wall and membrane-lined invagination called an infection thread (IT), which the rhizobia gain access to Murray (2011). As the IT extends through the epidermis and ultimately into the underlying cortex, bacterial cells close to the growing tip of the IT grow and divide, in effect enabling the bacteria to traverse the IT. Concomitant with IT formation, there is de-differentiation and division of underlying cortical cells, resulting in the formation of a nodule meristem (Patriarca et al., 2004). Rhizobia then internally colonize the plant root, first gaining access to the intracellular space, and then infecting cells of the nodule primordia.

Bacteria in the developing nodule primordia are enclosed within a host-derived membrane, obtained as they exit the infection thread, giving rise to an organelle called the symbiosome (Brewin, 2004). Bacteria in the symbiosome differentiate into specialized nitrogen fixing bacteroids, losing their ability to replicate in the process (Oke and Long, 1999). There is a distinction between legumes in which the meristem is transient (determinate nodulators) or maintained (indeterminate nodulators). The nodules of indeterminately-nodulating species are able to persist indefinitely whilst determinate nodules eventually senesce (Gibson et al., 2008). Many legumes forming indeterminate nodules belong to a clade termed the inverted repeat-lacking clade (IRLC) on the basis of the absence of one of two 25 kb inverted repeats in their chloroplast genome. In the case of nodules of IRLC legumes, bacteroids are almost always unable to resume vegetative growth should they be released from the nodule (terminal bacteroid differentiation, TBD). TBD is associated with more extreme cell enlargement and genome endoreduplication and is thought to lead to enhanced efficiency of N-fixation [reviewed in Alunni and Gourion, 2016]. Inside the nodule, bacterial nitrogenase breaks down atmospheric dinitrogen into ammonia which is provided to the host.

Incompatibility during legume-rhizobia symbiosis may manifest in different ways, depending on the stage at which it occurs. Whilst some legume-rhizobia pairings may not symbiotically interact at all [for instance, *M. truncatula* and *Mesorhizobium loti* (Radutoiu et al., 2007)], other ultimately incompatible pairings can progress through the very early stages of the symbiosis, such as inducing root hair curling, only to fail to colonize the root or form nodules (known as a *nod*^−^ outcome) e.g., *M. truncatula* F83005.5 and *Sinorhizobium meliloti* Rm41 (Liu et al., 2014). It is also possible that an interaction can result in normal nodule morphogenesis only for the resultant nodules to be either uninfected or deficient in nitrogen fixation (known as a *fix*^−^ outcome^−^) e.g., *M. truncatula* A17 and *S. meliloti* Rm41 (Liu et al., 2014). Even interactions resulting in nitrogen fixation may not represent maximum compatibility. For instance, the widely used model organism *S. meliloti* 1021 is a natural symbiont of *Medicago sativa* (alfalfa) but is also able to form *fix*^+^ nodules with some accessions of closely related *M. truncatula*. However, the efficiency of nitrogen fixation during the interaction with *M. truncatula* is substantially lower than natural *M. truncatula* symbionts such as *S. meliloti* 1022 or *Sinorhizobium medicae* 419 (Terpolilli et al., 2008; Kazmierczak et al., 2017).

Nodule formation incurs a significant cost for the host plant in terms of photosynthates to supply N-fixing nodules, and therefore an optimal strategy is to only participate in symbiosis with bacteria that fix nitrogen efficiently in return. However, a lifestyle closer to parasitism, sometimes termed “cheating” may be more beneficial from the perspective of the bacteria if it is able to sequester carbon, whilst providing little or no nitrogen fixation in return. Cheating is especially a consideration in some legume-rhizobia interactions where bacteroids are subject to TBD and therefore where the process of fixing nitrogen occurs a huge fitness cost to the rhizobial population as a whole (Denison and Kiers, 2004). It is possible that cheating happens more frequently in legume species where bacteroid differentiation is less well-advanced, since terminal bacteroid differentiation could be considered to enable a greater degree of host control. Studying a wider range of host-symbiont combinations across the legume phylogeny would certainly help to explore this interesting question.

Although N-fixing symbiosis is often considered in terms of an interaction between a legume and a single strain of rhizobia, the root is typically exposed to mixed populations of rhizobia in the rhizosphere. Therefore, it is crucial that the host is able to not only discern “friend” vs. “foe” (i.e., between a compatible symbiont and a cheater or a *bona fide* pathogen) but also between “friend” and “best friend” (i.e., between a poorly matched and an efficient symbiotic partner) in order to optimize N-fixation to satisfy the nutritional needs of the host. This review considers the mechanisms that legumes and rhizobia employ to identify each other, and how these can facilitate partner selection.

## Flavonoids as the Primary Determinant of Rhizobial Host Range

**Figure 1:**
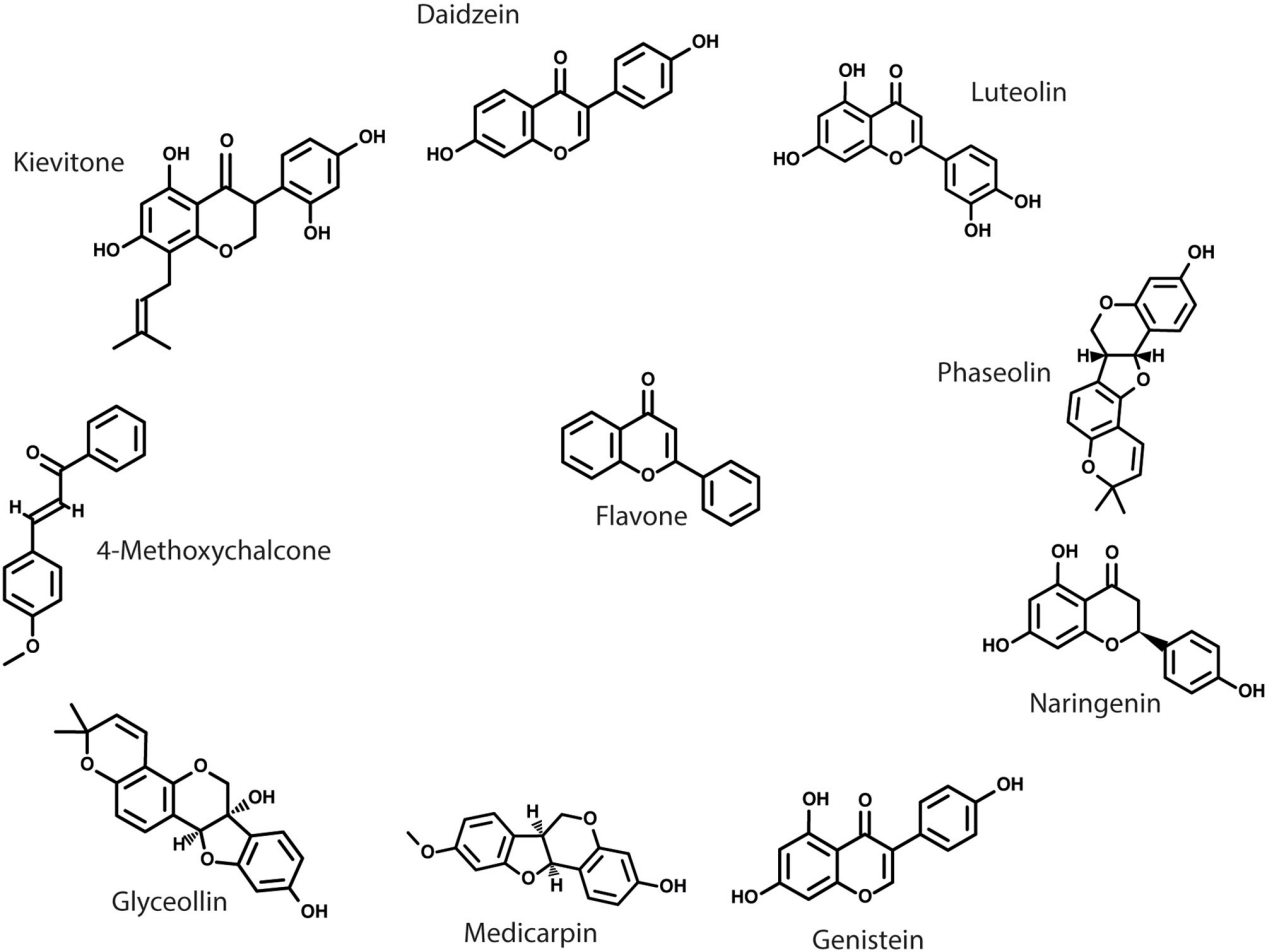
Structures of select flavonoids associated with legume nodulation. Flavonoids are plant secondary metabolites of plants that have many diverse functions. Many distinct flavonoids are found in plants, consisting of chemical modifications to a core flavone structure.

The initial signaling events during legume-rhizobia associations provide the first opportunity for partner choice by both parties. The first step of this exchange is the exudation of flavonoids from the root of the prospective host plant. Legumes possess an enormous diversity of flavonoids, although evidence so far suggests that only a subset of these are involved in symbiosis (Figure 1). Flavonoids are additionally responsible for many developmental and allelopathic processes in legume and non-legume plants alike [reviewed in Weston and Mathesius, 2013]. The presence of flavonoids may influence rhizobial host range by two mechanisms [reviewed in Liu and Murray, 2016]; either acting as an infection signal and stimulating rhizobial *nod* gene expression or acting as a phytoalexin and eliciting antimicrobial activity.

For a flavonoid to serve as an infection signal, it must not only possess the ability to elicit Nod factor production *in vitro* but must also be physically present in the root exudate. For instance, the flavonoid luteolin is a potent inducer of *nodD* expression in *S. meliloti* but is naturally absent from the root exudate of its natural host *M. sativa* (Maxwell et al., 1989). It has long been known that flavonoids that act as infection signals induce *nodD* activity only in specific species or strains of rhizobia (Spaink et al., 1987). There is strong evidence that methoxychalcone is the primary flavonoid infection signal in *M. sativa*/*truncatula* whilst genistein and daidzein are responsible for *nodD* activation in *Glycine max* (soybean)/*Bradyrhizobium* symbiosis [reviewed in Liu and Murray, 2016]. The role of flavonoids as a primary host range determinant has been elegantly demonstrated by expressing *nodD* from various donor strains in a strain of rhizobia with no endogenous functional *nodD* genes. Expression of *R. leguminosarum* bv. viciae and *R. leguminosarum* bv. trifolii *nodD* in *S. meliloti* strain A2105 (which has insertions inactivating all three of its endogenous copies of *nodD*) allows activation of *nod* gene expression in the presence of naringenin or 7-hydroxyflavone and, only in the case of bv. viciae, eriodyctiol (Peck et al., 2006).

Some flavonoid compounds show increased production following rhizobial inoculation but do not themselves activate *nod* gene expression, instead acting as antimicrobials. The release of these phytoalexin flavonoids specifically in response to rhizobia would suggest that they are still involved in legume-rhizobia symbiosis. The *Phaseolus vulgaris* symbiont *Rhizobium etli* is able to grow in the presence of some legume phytoalexin flavonoids, including kievitone and phaseolin, which were first isolated from its natural host. *S. meliloti* is able to tolerate medicarpin, which is found in the exudates of the compatible host *M. truncatula* (Pankhurst and Biggs, 1980). This would suggest that tolerance to antimicrobial flavonoids produced by a potential host is a prerequisite for symbiotic compatibility. Given that these flavonoids are still exuded into the rhizosphere, their presence is likely to have a significant effect on the community composition, which could, in the longer term, lead to formation of a niche for favored symbionts to thrive within. There is also evidence that resistance to flavonoids may sometimes be under the control of additional host flavonoids. For instance, glyceollin is toxic to the *G. max* symbionts *B. japonicum* and *Sinorhizobium fredii* but pre-incubation with genistein and daidzein induces resistance. The mechanism of this resistance is independent of *nod* gene activation by flavonoids because the effect is replicated in bacteria that do not have a functional *nodD* gene (Parniske et al., 1991).

Distinct legume species exude different combinations of flavonoids. By only recognizing a specific flavonoid profile, a rhizobial strain is able to reciprocate symbiosis signals only in the context of a compatible host. Moreover, the resistance of compatible strains to antibiotic flavonoids may create a niche within the rhizosphere where the compatible strain(s) can replicate in an environment of reduced community competition. Thus, flavonoids serve as a first mechanism for both host and rhizobia to find their favored partners. This mechanism may be reinforced by production of phytoalexins from the host plant that are able to suppress the growth of less compatible rhizobia.

## Regulation of Host Range by Rhizobial Nod Factors

The ability of the host plant to recognize Nod factors during the initial cross-talk between the two parties is also a determinant of host range as subsequent events depend on the activity of nodulation-related genes such as *NIN* (Vernié et al., 2015), which are themselves expressed in response to Nod factor signaling. Although Nod factors share the same basic structure of a chitooligosaccharide chain connected to a fatty acid, they can be extensively modified by the bacteria. This has given rise to exceptional diversity of Nod factors across rhizobial species (Long, 1996). Specifically, variations in the extent of chitooligosaccharide polymerization, the nature of the attached fatty acid and chemical substitutions at either terminus of the molecule [reviewed in Mergaert et al., 1997] allow for different rhizobial species to produce chemically distinct combinations of Nod factors.

In much the same way that the ability to recognize flavonoids is the primary rhizobial determinant of host range, the ability to recognize Nod factors is the primary determinant of symbiosis specificity from the perspective of the host. Transfer of Nod factor genes between rhizobial strains may allow the recipient to colonize natural hosts of the donor strain. For instance, strains of *R. leguminosarum* with extensive deletions in their symbiosis plasmids are unable to form nodules with their natural host *Trifolium repens*. Subsequent transfer of a plasmid bearing the *nod* genes from *S. meliloti* permits the formation of *fix*^−^ nodules with *M. sativa*, the natural host of the donor rhizobia (Debellé et al., 1988). Additionally, expression of the fucosyl-transferase encoded by the *nodZ* gene of *Bradyrhizobium japonicum* in *Rhizobium leguminosarum* permits the formation of *fix*^−^ nodules in the tropical legumes *Macroptilium atropurpureum* (siratro), *Glycine soja, Vigna unguiculata* and *Leucaena leucocephala* (López-Lara et al., 1996). These experiments show that the Nod factor structure is at least partially responsible for determining the range of plant species (or accessions) that a given rhizobia is able to associate with.

Strains of *S. fredii* possess the Nod factor repressor *nolR* as well as two functional copies of *nodD*. Wild type *S. fredii* HH103 is able to associate with *Lotus burtii* via crack entry infection, independently of infection thread formation (Acosta-Jurado et al., 2016). *nodD1* mutants fail to form nodules with *L. burtii*, whilst *nodD2* or *nolR* inactivation leads to an extension of host range to *L. japonicus* Gifu. Interestingly, both the *nodD2* and *nolR* mutants produced Nod factors in higher quantities than the wild type upon genistein exposure and infected both *L. burtii* and *L. japonicus* via infection threads rather than crack entry (Acosta-Jurado et al., 2019). Subsequent study has revealed that inactivation of *SyrM*, another transcription factor involved in regulation of early Nod gene expression, is also sufficient to replicate the *fix*^+^/infection thread-dependent phenotype in *L. japonicus* and *L. burtii* (Acosta-Jurado et al., 2020). *nolR* (Vinardell et al., 2004), *nodD2* (Acosta-Jurado et al., 2019) and *SyrM* (Acosta-Jurado et al., 2020) mutants all exhibit diminished ability to infect *G. max* relative to wild type HH103. Despite its broad potential host range, HH103 may therefore represent an example of a rhizobial strain that has evolved to restrict interactions with legumes that it is less well-suited to colonize, in favor of its natural host, *G. max*.

Nod factors are perceived by Nod factor receptors to instigate the symbiosis pathway and thus Nod factor receptors themselves represent an additional component of host range specificity The role of these receptors in symbiosis specificity has been demonstrated by transformation of *M. truncatula* with the *LjNfr1*/*LjNfr5* genes of *L. japonicus*. Inoculation of *M. truncatula Nfr1*^+^/*Nfr5*^+^ with strains of the *L. japonicus* symbionts *M. loti* or *R. leguminosarum* DZL modified to constitutively express NodD (thus producing Nod factors independently of flavonoid signaling) led to nodule formation, which does not occur in wild type *M. truncatula*. However, these nodules had a *fix*^−^ phenotype and their infection was arrested before symbiosome formation (and hence N-fixation) could occur (Radutoiu et al., 2007).

Recent work by Bozsoki et al. (2020), in which chimeric receptors comprising domains from *Lj*NFR1, the related chitin receptor *Lj*CERK6 and *Mt*LYK3 were generated, has led to a significant enhancement in our understanding of Nod factor recognition. These chimeric proteins were expressed in *nfr1 L. japonicus* mutants inoculated with *M. loti*, allowing the sensitivity of the engineered receptor to *M. loti* Nod factor to be assessed by the extent of nodule formation. Nod factor specificity of NFR1 was mapped to two regions within the LysM1 domain of the extracellular domain of the protein but the transmembrane and kinase domains were also found to influence the efficiency of nodulation. Substitution of residues associated with Nod factor recognition in the LYK3 LysM1 domain with corresponding residues from NFR1 still permitted nodulation of *nfr1* plants. Similarly, expression of a chimeric receptor with the NFR1 LysM1 domain but substituting the Nod factor recognition regions with those of LYK3 (and an additional region of the LYK3 LysM1 domain) allows recognition of *S. meliloti* by *lyk3 M*. *truncatula*. Interestingly, receptors combining the CERK6 ectodomain with the NFR1 transmembrane and kinase domains still permitted nodulation of *nfr1* plants, provided the ligand specificity regions of the LysM1 domain were substituted with corresponding regions from NFR1 (Bozsoki et al., 2020).

## Recognition of Rhizobial Polysaccharides Underpinning Symbiotic Interaction

Extracellular polysaccharides produced by rhizobia, such as exopolysaccharides (EPS) and lipopolysaccharides (LPS), are indispensable during many legume-rhizobia interactions due to their roles in root attachment, signaling and the suppression of host immunity [reviewed in Downie, 2010]. The identification of the *L. japonicus* receptor kinase *Epr3* and the demonstration that it directly binds to and enables perception of the EPS of the compatible symbiont *M. loti* R7A (Kawaharada et al., 2015) has suggested that rhizobial exopolysaccharides moderate symbiosis by regulating receptor-ligand interactions. Given that there is extensive diversity in polysaccharide structure across rhizobia species, it is therefore plausible that specificity in polysaccharide recognition may represent an additional aspect of host range regulation, akin to Nod factor recognition.

The exopolysaccharide succinoglycan of *S. meliloti* is required for infection of its natural host *M. sativa* (Cheng and Walker, 1998) and in interactions with compatible accessions of *M. truncatula* (Simsek et al., 2007; Liu et al., 2014). Transfer of a segment of the succinoglycan-coding *exo* gene from the A17-compatible *S. meliloti* 1021 into the usually incompatible Rm41 strain results in a *fix*^+^ phenotype in A17 that is comparable to the 1021 donor strain (Simsek et al., 2007). In addition to its role in facilitating infection during the early stages of the symbiosis, there is also evidence that succinoglycan acts to protect *S. meliloti* from the bactericidal effects of NCR247, a peptide belonging to the nodule-specific cysteine-rich (NCR) family of peptides (discussed later in this review) during later stages of symbiosis. Both overexpression and exogenous application of succinoglycan leads to dramatically enhanced survivability of cells in the presence of otherwise toxic concentrations of NCR247 (Arnold et al., 2018).

A possible role for LPS in symbiosis specificity is supported by observations of the broad host range *S. fredii* strain HH103. This strain is able to form nodules with many legume species including its natural host *G. max, Cajanus cajan* (pigeon pea) and IRLC member *Glycyrrhiza uralensis* (liquorice) (Crespo-Rivas et al., 2016). Remarkably, in the latter example, the endoreduplication and poor external survivability of bacteroids, which is seen as a hallmark of TBD in nodules of clade members, is absent. Whilst LPS signatures of HH103 bacteroids isolated from non-IRLC members *G. max* and *C. cajan* nodules do not exhibit alterations relative to free-living bacteria, modifications were observed in bacteroids isolated from *G. uralenis* nodules (Crespo-Rivas et al., 2016). Further study is needed to clarify the contribution of these LPS modifications to the unusual absence of terminal differentiation seen in this interaction.

## Strain-Specific Restriction by Effector-Triggered Immunity

Effector-trigger immunity (ETI) is a layer of plant innate immunity directed against effector proteins used by microorganisms to enhance virulence or circumvent host immunity. Resistance (R) proteins are receptors that are responsible for either recognizing pathogen effectors directly or detecting modification to endogenous proteins as a result of the activity of pathogen effectors [reviewed in Cui et al., 2015]. Many pathogens use a specialized apparatus called a secretion system to translocate effector proteins directly into the cytoplasm of host cells. Some rhizobia are also known to use type III or IV secretion systems (T3SS/T4SS) to deliver effector proteins into target cells to aid infection [reviewed in Soto et al., 2009]. Delivered rhizobial effectors have been found to have both positive and negative effects on symbiosis, often depending on the species or accession of the host. For instance, *Bradyrhizobium* sp. DOA9 (Songwattana et al., 2017) and *M. loti* MAFF303099 (Okazaki et al., 2010) mutants deficient in protein secretion show an increased ability to form nodules with some hosts but decreased ability with others. Meanwhile, knockout of *Bradyrhizobium* sp. ORS3257 effectors leads to enhanced nodulation or symbiotic defects in *Aeschynomene indica* depending on the specific effector (Teulet et al., 2019). It has been theorized that some legumes may have evolved R proteins that detect specific rhizobial effectors and activate defense responses to prevent colonization, thus serving as another mechanism of control of host range.

The role of ETI during symbiosis has been best studied in soybean (*G. max*) which possesses at least eight known genes involved in strain specific restriction of nodule formation (Hayashi et al., 2012). Amongst these, the dominantly-acting *Rj2, Rfg1* and *Rj4* genes have been best characterized and are involved in restricting symbiosis with certain strains of *Bradyrhizobium* spp. and *S. fredii*. More specifically, *Rj2* and *Rfg1* are classical R proteins that have been shown to restrict nodulation by *B. japonicum* USDA122 or *S. fredii* USDA257, USDA205 and USDA193 respectively (Yang et al., 2010; Fan et al., 2017). Inactivation of the T3SS of USDA122 permits the formation of functional N-fixing nodules in the previously incompatible wild type Hardee accession (Tsukui et al., 2013), suggesting that ETI likely precludes symbiosis in the wild type strain and that effector secretion is dispensable for symbiosis. The active variant of the *Rj4* allele is inferred to be an antimicrobial thaumatin protein rather than a classical R protein and prevents nodulation by *B. japonicum* Is-34 and *B. elkanii* USDA61. Through the employment of transposon insertions, the genetic basis of this incompatibility has been mapped to an inferred T3SS effector in *B. japonicum* Is-34 (Tsurumaru et al., 2015) and a region containing six genes in USDA61, including one with homology to a known *Xanthomonas campestris* pathogen effector (Tang et al., 2016). A role for T3SS activity in *Rj4-*USDA61 incompatibility is further supported by the finding that the expression of defense-related genes in the incompatible BARC-2 accession induced by wild type USDA61 is abolished in a strain with a non-functional T3SS (Yasuda et al., 2016).

Although the role of ETI during N-fixing symbiosis is best studied in soybean, there is evidence that this is used as a means of symbiont selection in other legume species. In addition to its incompatibility with accessions of soybean carrying the *Rj4* allele, *B. elkanii* USDA61 also interacts poorly with some accessions of mung bean (*Vigna radiata*). Five genes have been identified (*innA*-*E*) in USDA61 that are associated with *V. radiata* incompatibility. Knockout of any one these is sufficient to restore symbiotic compatibility with the *V. radiata* accession KPS1. Remarkably, the knockout of four of these genes was also sufficient to allow nodulation of *G. max* BARC-2 (Nguyen et al., 2017), suggesting that a common mechanism of symbiont restriction is conserved between the two legume species. The fifth gene, *innB*, encodes a T3 effector which is induced by genistein. Given that *innB* does not interfere with nodulation in *G. max* and positively regulates nodulation in the closely related *V. mungo* (Nguyen et al., 2018) it is likely that specific accessions of *V. radiata* possess an R protein directed against *innB* which precludes symbiosis with this strain.

A recent study of the ability of USDA61 to infect *Lotus* species has also implicated the T3SS of this strain as a source of incompatibility in *L. japonicus* and *L. burtii*. Inoculation using a mutant USDA61 strain that is deficient in effector secretion led to significantly enhanced infection of nodules, although infected nodules exhibited early senescence-like responses regardless. By mutating specific T3 effectors, *nopF* was identified as being responsible for inhibition of infection in *L. japonicus* Gifu whilst *nopM* was found to affect early nodulation senescence in *L. burtii* and the Gifu and MG20 accessions of *L. japonicus*. A third effector, as yet unidentified, is likely to prevent nodule maturation in *L. burtii* and *L. japonicus* Gifu. This suggests that some *Lotus* species and accessions employ ETI to prevent symbiosis with *B. elkanii* USDA61, including *L. japonicus* MG20, which has at least three ETI-based checkpoints to reinforce its incompatibility with USDA61 (Kusakabe et al., 2020).

Less is known about a potential role for ETI in symbiont selection in leguminous species that form indeterminate nodules (such as *M. truncatula*). One reason for lack of study here is the knowledge that the NCR family possessed by IRLC members within this group already exhibits substantial influence over symbiont compatibility, as described later. By monitoring compatibility between *S. meliloti* Rm41 and different accessions of *M. truncatula*, Liu et al. (2014) have identified a locus containing eight genes associated with enabling nodulation specificity. Experiments crossing the *fix*^+^ A20 accession with *nod*^−^ F83005.5 suggest that this specificity is regulated by a single dominant gene, termed *NS1*, and thus it is possible that this represents an example of ETI. However, Rm41 is not known to possess a T3SS and the precise nature of the gene underpinning this phenotype remains unknown (Liu et al., 2014).

ETI provides a mechanism by which legume species could be able to restrict interactions with less favored rhizobial strains with exquisite specificity. Because ETI depends on both host (R proteins) and rhizobial (effector proteins) factors, it is often the basis of incompatibility between specific legume accessions and rhizobial strains. Secreted effector proteins used by the rhizobia to aid infection make ideal targets for ETI because they cannot easily escape recognition by mutation without losing their activity. Given the resemblance of the role of ETI during legume-rhizobia symbiosis to what occurs during some pathogen interactions, it could be speculated that rhizobial strains targeted by ETI in this way could interfere with R-gene mediated recognition of their effectors through the use of additional effectors and thus continue to infect a legume host, although this has not yet been demonstrated.

## Regulation of Rhizobial Host Range by Nodule-Specific Cysteine-Rich (NCR) Peptides in the Inverted Repeat Lacking Clade (IRLC) of Legumes

Nodules belonging to the inverted-repeat lacking clade (IRLC) of legumes are marked by more extreme bacteroid differentiation, and this is mediated, at least in part, by antimicrobial peptides belonging to the nodule-specific cysteine-rich (NCR) family (Van de Velde et al., 2010; Roy et al., 2020). The role of NCR peptides is best understood in *M. truncatula*, which has over 700 inferred NCRs to date (Maróti et al., 2015), although it is unclear if all NCR family members are involved in regulating symbiosis since their expression levels and pattern can vary [reviewed in Roy et al., 2020]. Patterns of NCR peptide expression vary greatly between nodules of *M. truncatula* accessions but show little variation within accessions in response to different strains of rhizobia (Nallu et al., 2014). Given their large number and that the expression of NCR peptides is not tailored to the symbiont, expression of NCR family members is an ideal means to discern between potential symbiotic partners alongside their role in enforcing TBD of nodule-resident rhizobia.

In *M. truncatula*, certain NCR peptides have been demonstrated to have negative effects on the viability of specific rhizobial strains within nodules. *S. meliloti* strain Rm41 is able to infect and form nodules in both the DZA315 and A17 accessions of *M. truncatula* but a *fix*^−^ phenotype is observed in the latter case. The basis for this incompatibility with A17 has been mapped to two loci, named *NFS1* (Yang et al., 2017) and *NFS2* (Wang et al., 2017), which both encode NCR peptides. The peptide sequences of NFS1 and NFS2 in *M. truncatula* A17 differ by one and three amino acid substitutions, respectively from the corresponding DZA315 sequences. In either case, the A17 isoform of the peptide exhibits antimicrobial activity against Rm41 *in vitro*. However, this is not sufficient to explain the *fix*^−^ phenotype in A17; the DZA315 variant of NFS1 is also bactericidal against Rm41 (Yang et al., 2017) yet this pairing still results in N-fixing symbiosis. Furthermore, A17 is able to form *fix*^+^ nodules with *S. medicae* strain ABS7, despite this strain also being susceptible to A17 NFS2 *in vitro*. Knockout of *NFS1* is also sufficient to allow Rm41-infected nodules to fix nitrogen in A17 plants that still possess a functional copy of *NFS2* (Wang et al., 2017). Given that knockout of the A17 variants of *NFS1*/*NFS2* results in *fix*^+^ nodules, the role of the DZA315 variants of these NCRs is unclear. The *S. meliloti* strain A145 also forms *fix*^+^ nodules with DZA315 and *fix*^−^ nodules with A17, with the A17 variant of the *NFS1*/*NFS2* genes acting dominantly to preclude nitrogen fixation with strain A145 (Wang et al., 2018). Therefore, some isoforms of NCR peptides appear to restrict symbiosis with specific rhizobial strains.

Despite the antimicrobial activity of many NCR peptides, the expression of some NCR family members is essential for symbiosis between *M. truncatula* and some rhizobial strains. Knockout of the *M. truncatula* gene encoding NCR211 results in plants that are symbiotically ineffective in partnership with the normally compatible *S. meliloti* 1021 (Kim et al., 2015). Although infected plants are still able to form nodules, the resulting organs fail to elongate and fix nitrogen, despite expression of bacterial *nif* genes and normal accumulation of leghemoglobin. Bacteria within mutant nodules are rarely able to fully differentiate and are unable to persist intracellularly (Kim et al., 2015), suggesting that NCR211 is required for long-term rhizobial viability within nodules. Similarly, perturbing the expression of *M. truncatula* NCR169 also interferes with nodule viability following infection with *S. meliloti* 1021 or *S. medicae* 419. Substitution of any of the four cysteine residues present in the mature NCR169 peptide sequence is sufficient to produce a *fix*^−^ phenotype (Horváth et al., 2015). Given the large size of the NCR family in *M. truncatula*, the finding, in two distinct cases, that removal of a single peptide is sufficient to abolish successful symbiosis is remarkable. Despite being so numerous, some NCR peptides are clearly not functionally redundant and do not simply influence symbiosis on the basis of their antimicrobial activity.

There is strong evidence to suggest that rhizobial tolerance of NCR peptides depends on the activity of BacA and BacA-like proteins. These are membrane transport proteins that have been found to be essential for rhizobia to survive within the symbiosome of legume species belonging to the IRLC (reviewed in Roy et al., 2020), although their presence in rhizobia that do not interact with IRLC legumes and also many other bacteria besides, indicates they are likely to have functions outside of symbiosis. Deletion of the *bacA* gene of *S. meliloti* Rm2011 alone is sufficient to result in a *fix*^−^ phenotype in previously compatible nodules of *M. sativa* and *Melilotus alba*. In *M. sativa*, a *fix*^+^ phenotype cannot be recovered by complementation of Rm2011 *bacA* mutants with the *bacA* gene of *R. leguminosarum* bv. viciae 3841 or *S. fredii* NGR234. In contrast, expression of either the 3841 or the NGR234 *bacA* genes under the native Rm2011 promoter leads to a *fix*^+^ phenotype in which the extent of N-fixation was comparable to wild type Rm2011 or roughly half, respectively (diCenzo et al., 2017). These results suggest that the *bacA* gene of *S. meliloti* has evolved to interact specifically with *M. sativa*. This is supported by phylogenetic analysis indicating that the *bacA* gene of *S. meliloti* Rm2011 has undergone rapid evolution and its sequence now resembles the *bacA* gene of pathogenic genera *Klebsiella, Brucella* and *Escherichia* more closely than it resembles many other rhizobial *bacA* orthologs (diCenzo et al., 2017). This suggests that BacA and BacA-like proteins possessed by rhizobia may be a determinant of host range when infecting legumes belonging to the IRLC and that this is likely mediated by interactions with host NCR peptides.

Taken together, the above data suggest that NCR peptides have roles in both encouraging symbiosis with favorable partners and restricting symbiosis with less favored rhizobia. However, it is possible that some rhizobial strains may have evolved mechanisms to interfere with the activity of NCR peptides, thus providing them with a means of moderating their own host range. *S. meliloti* strain B800 is able to form *fix*^+^ nodules in *M. truncatula* accession A17 but not A20, with the latter outcome dependent on the expression of the pHRB800 accessory plasmid possessed by the bacteria (Crook et al., 2012). More specifically, this phenotype has been mapped to the activity of a single gene on the plasmid, the peptidase *hrrP*, the expression of which results in enhanced bacterial proliferation in both A17 and A20 nodules. Given that this peptidase has been demonstrated to cleave some NCR peptides *in vitro*, it is likely that it interferes with the activity of host NCR peptides and thus their effects on the proliferation and differentiation of nodule-resident rhizobia. The formation of *fix*^−^ A20 nodules is likely a side effect of this (Price et al., 2015). This suggests a mechanism by which less-favored rhizobial strains or even cheaters are able to colonize hosts by directly targeting a host mechanism of control of symbiont selection.

The finding that certain components of *S. meliloti* EPS provide protection against the antimicrobial effects of NCR247 (Arnold et al., 2018) may serve as another mechanism by which rhizobia may resist moderation of their differentiation by their hosts. It is currently unclear if this is specific to NCR247, or if this mechanism provides a more generalized defense against NCR family activity. There is also an association between polysaccharide alterations and the absence of TBD observed during *S. fredii* HH103-*G. uralensis* symbiosis. Given the relatively low sensitivity of HH103 to *M. truncatula* NCR247 and NCR335 (Crespo-Rivas et al., 2016) and the small number of known NCRs (seven) possessed by *G. uralensis* (Montiel et al., 2017), it is possible that blanket resistance to NCR activity provides a mechanism by which HH103 is able to escape imposition of TBD by its host plant during this interaction. Further research is required to address this question and the role, if any, of LPS modifications in it.

Despite their relatively recent discovery, there is now an abundance of evidence that NCR peptides are key determinants of symbiont compatibility in *M. truncatula* and likely other members of the IRLC of legumes. Although NCR peptides are thought to mediate TBD by interfering with regulators of the bacterial cell cycle (Mergaert, 2018), little is known about their targets, aside from NCR247 (Farkas et al., 2014; Penterman et al., 2014). Therefore, for the NCR peptides which promote symbiosis with select strains, identifying their bacterial targets would provide great insight into their activity. Given the exceptional number (hundreds) of NCR family members possessed by some IRLC species, it is likely that that specificity in bacterial targets is one component that has enabled diversification of NCR family members. This also allows symbiotic compliance to be reinforced by multiple mechanisms throughout the symbiosis. Given the fast-evolving nature of this area of research, it is hoped that the functions of other NCR family members outside *M. truncatula* will also be elucidated in the near future.

## Host Sanctions on the Basis of Nitrogen Fixation Efficiency

**Figure 2:**
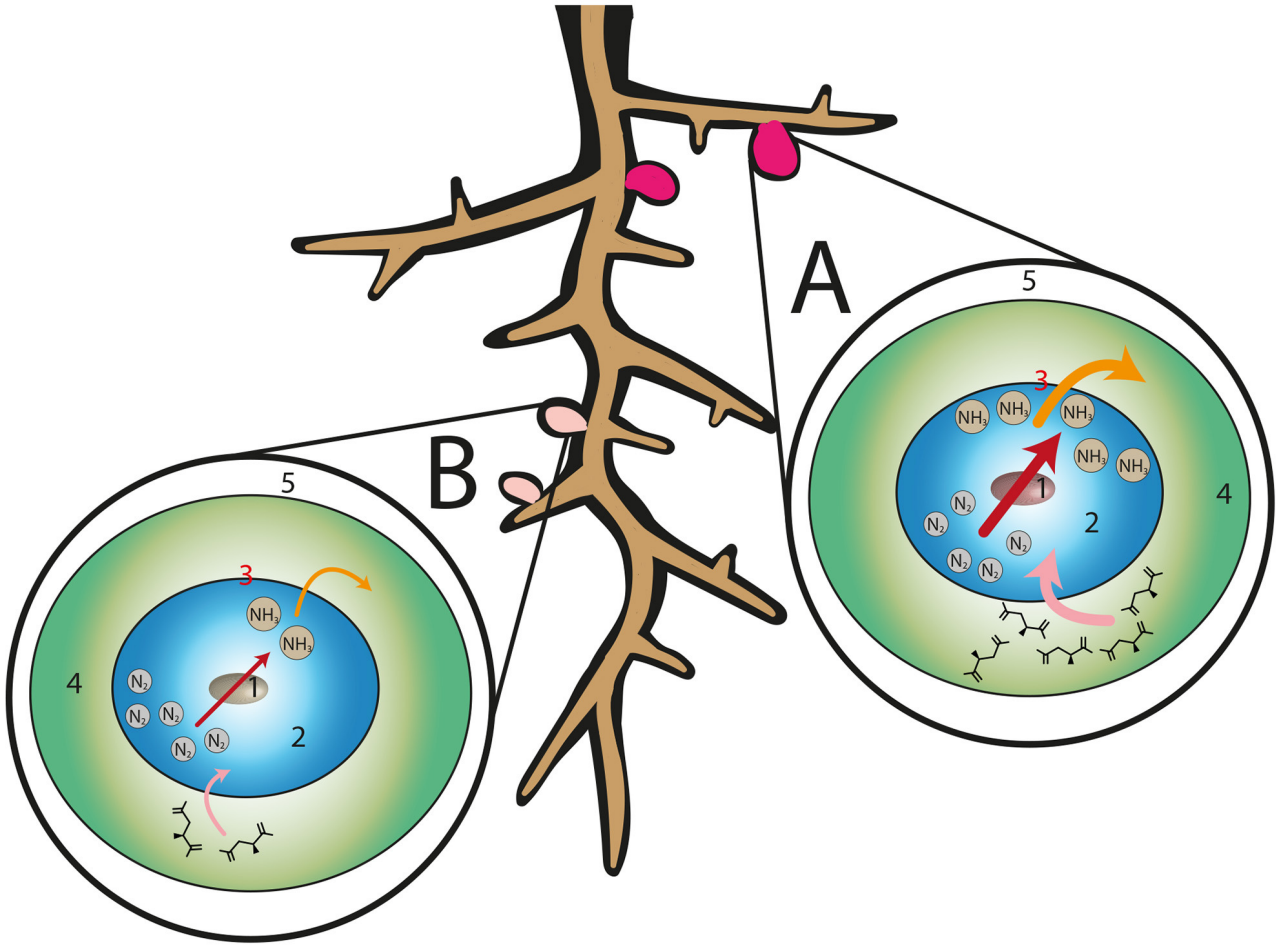
Host sanctioning in legume-rhizobia symbiosis. In the event of a compatible plant-rhizobia interaction, root nodules can be colonized by rhizobia that have different N-fixation efficiencies. For example, nodules may be colonized by rhizobia that are **(A)** high efficiency and thus favored from the perspective of the host plant, or **(B)** poorly matched but not incompatible. Inside root nodules **(A,B)** atmospheric nitrogen is converted to ammonia by symbiosome-resident rhizobia (red arrows) which is then transferred to the host plant (orange arrows). Photosynthates are provided to rhizobia residing in nodules in the form of malate (pink arrows). In the event that a nodule contributes little or no nitrogen fixation **(B)** the host plant may deprive the offending nodule of resources, such as photosynthates, to impede the development of that nodule. Labels: 1–bacteroid, 2–peribacteroid space, 3–peribacteroid membrane, 4–cytosol, 5–infected cell.

Although symbiont selection generally occurs during initial rhizobia-legume interactions, some legumes also possess mechanisms to discriminate between symbionts after colonized nodules have formed. In a natural rhizosphere ecosystem a root nodule will likely contain mixed populations of rhizobia in addition to other symbionts, parasites and other commensals [reviewed in Martínez-Hidalgo and Hirsch, 2017]. Whilst co-inoculation experiments suggest that legumes form more and larger nodules with preferred rhizobia (Heath and Tiffin, 2009), whether or not the plant actively penalizes nodules that do not fix nitrogen efficiently is less clear. In this context, sanctions are distinct from partner selection. Partner selection describes the preferential formation of nodules with a particular strain of rhizobia from a population of multiple compatible strains (and may be facilitated by any of the previously discussed mechanisms), whilst host sanctioning describes a mechanism to discriminate between and regulate the function of nodules (such as preferentially allocating photosynthates to productive nodules) once symbiosis is already established (Figure 2). Such control could impact the viability of rhizobia in these nodules and thus allow underperforming or cheating rhizobia strains to be “punished” by the host plant.

Host sanctions were first demonstrated by exposing nodules of *G. max* infected with *B. japonicum* to a modified atmosphere in which nitrogen was replaced with argon, thus preventing nitrogen fixation. Bacterial proliferation was dramatically reduced in plants, roots and even individual nodules exposed to the nitrogen-free atmosphere, and this was apparently mediated by reducing the oxygen permeability of offending nodules (Kiers et al., 2003). The approach of eliminating atmospheric nitrogen from nodules has also been used to demonstrate sanctions in *P. sativum* and *M. sativa*, which form indeterminate nodules (Oono et al., 2011).

The above experiments do suggest that some legumes have the ability to sanction the occupants of ineffective nodules. However, these studies occurred in an artificial environment in which BNF is almost completely eliminated. Other studies (with active BNF) have found evidence for partner selection but not sanctions in the *M. truncatula*-*S. meliloti* mutualism (Heath and Tiffin, 2009; Gubry-Rangin et al., 2010). Additionally, during some legume-rhizobia interactions that provide only low levels of N-fixation, such as *M. truncatula* and *S. meliloti* 1021, infected nodules persist regardless (Terpolilli et al., 2008). There are a number of possible explanations for this; firstly, sanctions may not be universal amongst all legumes. Secondly, further work in *G. max* suggests sanction severity inversely correlates with the extent of N-fixation, and as such, rhizobia that contribute even small amounts of N-fixation could escape the strongest sanctions (Kiers et al., 2006). Thirdly, some rhizobia may possess mechanisms to avoid host sanctioning despite performing poorly with regards to N-fixation. Finally, previous experiments that did not find evidence for host sanctioning did so by comparing non-isogenic strains of rhizobia (Heath and Tiffin, 2009; Gubry-Rangin et al., 2010). These strains would likely exhibit differences, besides their ability to fix nitrogen in a given host, that influence their ability to colonize the host and this may have confounded the results of these studies. This last point has been convincingly addressed by Westhoek et al. (2017) who infected *P. sativum* with a strain of *R. leguminosarum* with a disrupted *nifH* gene. This mutant strain was therefore unable to participate in BNF but was otherwise identical to its parental strain. The authors assessed if the host plant was able to discern between the parental *fix*^+^ strain and the *nifH* mutant (and thus exercise partner choice), by co-inoculating plants with both strains, each possessing a distinct marker gene allowing them to be distinguished by staining, to visualize rhizobial presence in nodules. The proportion of nodules infected with the *fix*^+^ strain accurately reflected the proportion of the inoculum made up by this strain, demonstrating the absence of partner choice between the two strains by the host plant. However, nodules infected with the *nifH* mutant *fix*^−^ strain were significantly smaller than those infected with the wild type strain. This provides further evidence that *P. sativum* is able to penalize poorly performing nodules and consequentially is capable of sanctioning although the effect of any sanctions on the fitness of rhizobia within any sanctioned nodule remains unclear (Westhoek et al., 2017).

From an evolutionary perspective, it can be considered preferable for a host plant to accommodate the most efficient rhizobia (in terms of N-fixation) present in its surroundings; termed partner choice. This requires the compatibility of a putative symbiont to be assessed prior to the onset of N-fixation, and this could be mediated by a combination of the signaling factors discussed previously (for instance, Nod factor recognition and ETI). In situations where differences between rhizobial strains may not be perceivable by the host, e.g., in the experiments carried out by Westhoek et al. (2017), host sanctioning could provide an additional layer of security that is much harder for less efficient rhizobia to cheat. Outstanding questions relating to sanctions include how the N-fixing contribution of individual nodules is assessed, and how (or if) sanctioning occurs in nodules with mixed populations of rhizobia of varying N-fixation efficiency where the extent of N-fixation is likely to be intermediate. In such an instance, the absence of sanctioning would allow cheaters to thrive but tightening of sanctions would punish those individual rhizobia which are delivering optimal N-fixation.

## Transfer RNA-derived Small RNA Fragments Provide a Novel Mechanism by which Rhizobia Can Manipulate Host Gene Expression

The identification of transfer RNA (tRNA)-derived small RNA fragments (tRFs) involved in the regulation of N-fixing symbiosis provides another mechanism by which host specificity in the legume-rhizobia symbiosis is likely enabled. tRFs are generated by cleavage of tRNAs at specific regions, giving rise to small RNAs that may be able to silence the expression of target genes in a manner analogous to microRNAs (Sobala and Hutvagner, 2011). Ren et al. (2019) identified 25 distinct tRFs produced by *B. japonicum* USDA110 and inferred 52 putative targets of these in the soybean genome. Of these, three tRFs were found to suppress the expression of five host (soybean) genes that were putative homologs of proteins involved in root hair development in *A. thaliana*. Abolishing expression of these tRFs or overexpression of their targets resulted in attenuation of root hair curling and reduced nodule formation. Conversely, increased nodulation was observed if the host target genes were mutated, suggesting these host genes are negative regulators of nodulation. These tRFs were demonstrated to associate with the soybean ARGONAUTE-family protein GmAGO1b (Ren et al., 2019), suggesting they masquerade as host small RNAs and hijack the host RNA interference machinery to achieve silencing of target genes (as is a known function of the ARGONAUTE family, [reviewed in Mallory and Vaucheret, 2010].

Ren et al. (2019) also investigated conservation of this silencing mechanisms amongst other rhizobia and legumes. No variation was found in any of the three tRF sites in the eight *B. japonicum* strains tested or in the binding sites of their five target genes in 699 *G. max* accessions. This would suggest that this mechanism is universal in *B. japonicum*-*G. max* symbiosis. Of the target genes that had orthologs in *M. truncatula, P. vulgaris* or *L. japonicus*, the tRF target sites were absent or the corresponding tRFs were not known to exist in any compatible symbionts. However, the authors did infer the existence of ten *R. etli* tRFs that are predicted to target 14 host (*P. vulgaris*) genes (Ren et al., 2019). Therefore, it is likely that other rhizobial strains also use tRFs to control the expression of host genes, which would otherwise antagonize nodulation, in order to aid infection.

This novel silencing mechanism provides a remarkable example of gene expression within a legume host being directly molded by a rhizobial signal to promote symbiosis. In order to affect mRNA expression of host plant genes, rhizobial tRFs must be exported by an unknown mechanism to target cells where they interact with host machinery (Baldrich and Meyers, 2019). Given the recent nature of these findings, other examples of moderation of host gene expression by tRFs are likely to exist that have not yet been identified. If this silencing mechanism is conserved in other legume-rhizobia interactions, the diversity of tRFs possessed by rhizobial strains may also represent a broader additional layer in the regulation of host range during symbiosis. Another outstanding question is whether the plant host is itself able to influence rhizobial symbionts using similar mechanisms. Given that examples of both host-to-parasite and parasite-to-host trans-kingdom RNA signaling are now being found in plant-pathogen interactions [reviewed in Zhao and Guo, 2019], this possibility cannot be excluded.

## Concluding Remarks

**Figure 3:**
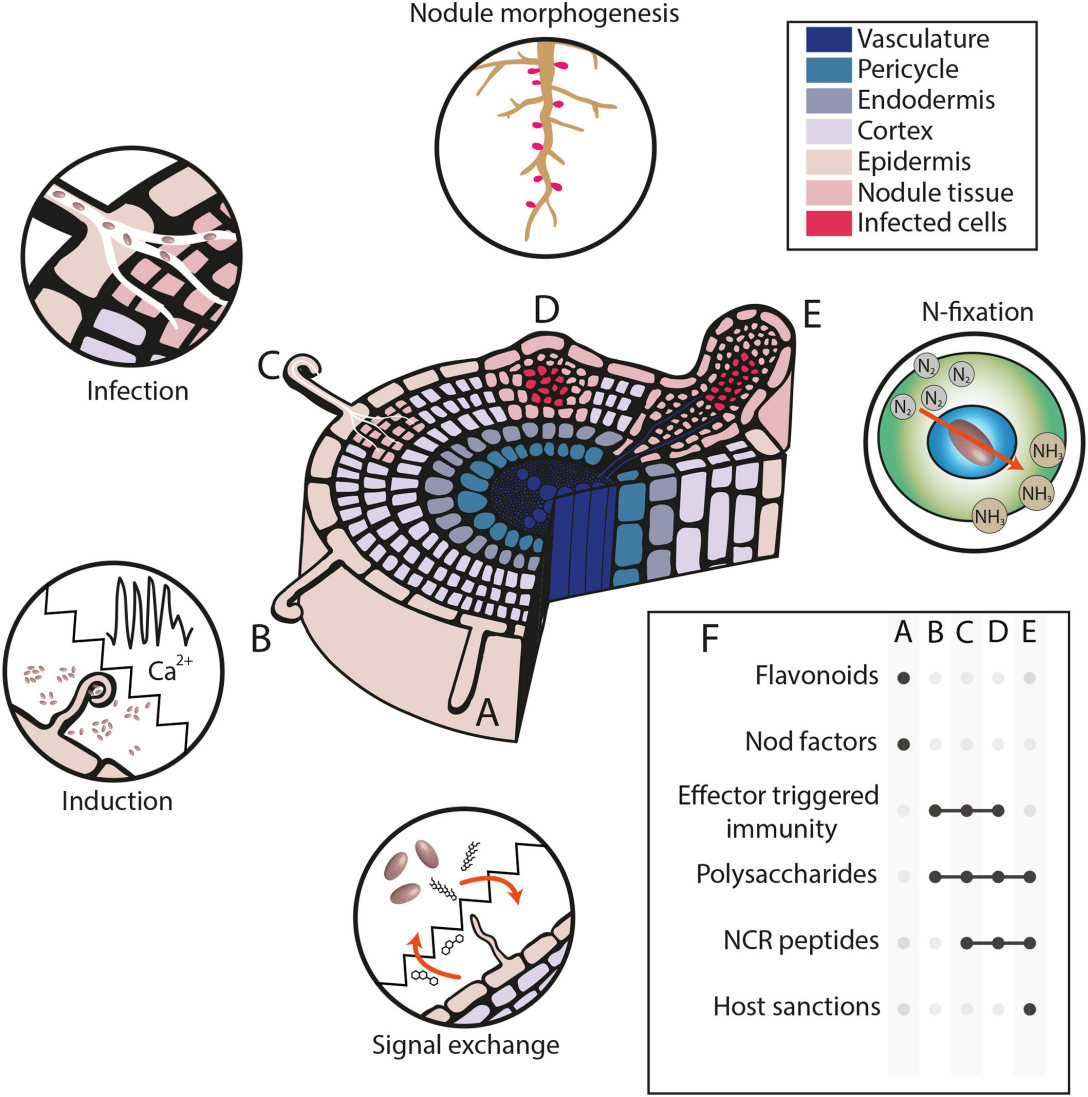
Determinants of host range specificity in legume-rhizobia symbiosis. **(A–E)** Cross-section of a legume root depicting typical stages of nodule formation during a compatible rhizobial interaction. **(A)** Flavonoids are exuded into the rhizosphere by the legume and are detected by rhizobia which reciprocate through Nod factor secretion. **(B)** Nod factor recognition induces changes in the plant including fluctuations in Ca^2+^ and curling of root hairs which can trap nearby rhizobia. **(C)** Rhizobia are able to invade the plant root through the formation of an infection thread; simultaneously, cell divisions in the cortex give rise to a nodule meristem. **(D)** The developing nodule enlarges due to continued cell divisions and its cells are colonized by rhizobia. **(E)** Within colonized cells, rhizobia are enclosed within specialized structures called symbiosomes where they differentiate into bacteroids and convert nitrogen obtained from the atmosphere into ammonia which is used by the host plant. **(F)** Summary of factors that influence host compatibility at each of the previously described stages, with stages highlighted with a black dot where a factor is a key determinant.

Compatibility between plant and rhizobial pairings during legume-rhizobia symbiosis is determined by many factors deriving from or being expressed in both host and symbiont. Both parties have evolved sophisticated mechanisms to recognize one another amongst the diversity of plant and bacterial life in the soil (Figure 3).

Compatibility may be reinforced at multiple stages throughout the symbiosis–for instance *S. meliloti* Rm41 is able to fix nitrogen with some accessions of *M. truncatula*, but whilst it is able to induce nodule organogenesis on A17 roots, resulting nodules fail to fix nitrogen and senesce early (Liu et al., 2014). At least two mechanisms underlie this incompatibility; the succinoglycan of this strain does not appear to correctly promote infection in A17 (Simsek et al., 2007) and the isoforms of NCR peptides NFS1/NFS2 in this accession preclude symbiosis (Wang et al., 2017; Yang et al., 2017). Interestingly, a *fix*^+^ phenotype is observed when either expressing a compatible succinoglycan or when *NFS1* is knocked out, which would suggest that neither mechanism is insufficient to constrain symbiosis by itself. Another interesting example of host-rhizobial control is provided by *S. fredii* HH103 which is normally associated with legumes that form determinate nodules, such as *G. max*. The early Nod gene expression of *S. fredii* HH103 seems to inhibit symbiosis with *Lotus* spp. (Acosta-Jurado et al., 2019), whilst this same strain is capable of symbiosis with IRLC member *G. uralensis*. *S. fredii* HH103 possesses a mechanism to escape TBD imposition by *G. uralensis*, possibly on the basis of modifications to its LPS (Crespo-Rivas et al., 2016).

This complexity of legume-rhizobia compatibility has implications for engineering symbioses (Pankievicz et al., 2019). Past attempts to transfer signaling components between rhizobia or legumes have allowed infection or even nodule formation between previously incompatible hosts. However, in spite of this, the resulting symbioses rarely result in nitrogen fixation and symbiosis is often terminated at a later stage (e.g., Debellé et al., 1988; López-Lara et al., 1996; Radutoiu et al., 2007). This supports the notion that host range is not simply based on compatibility in molecular dialogue pre-infection, but is instead reinforced at multiple steps throughout the symbiosis. Therefore, if nodulation is to be transferred to other plant species, a complete understanding of the determinants of symbiotic compatibility is necessary to optimize nitrogen fixation.

Currently, much of our understanding about host range determination is derived from approaches based on rhizobial genetics, due to the relative ease of genetically manipulating bacteria. Whilst this has been invaluable for our understanding of how partner selection occurs before and during infection, much more research is needed into the processes that govern symbiont compatibility in the later stages of symbiosis. Amongst the outstanding questions that need to be addressed is the prevalence of ETI and host sanctioning and the role of NCR peptides in IRLC legume species besides *M. truncatula*. Additionally, the mechanisms that facilitate symbiont selection against compatible but inefficient rhizobia merit further study. Together this will enable a more complete understanding of how host range of nitrogen fixing rhizobia is controlled which could help engineer rhizobia for use as agricultural inoculants or symbiotic partners for non-legumes.

## Author Contributions

All authors listed have made a substantial, direct and intellectual contribution to the work, and approved it for publication.

## Conflict of Interest

The authors declare that the research was conducted in the absence of any commercial or financial relationships that could be construed as a potential conflict of interest.
